# Impulsivity and psychiatric comorbidity as risk factors for suicide attempts in borderline personality disorder

**DOI:** 10.4102/sajpsychiatry.v28i0.1544

**Published:** 2022-05-30

**Authors:** Eman Shorub, Abdel Nasser Omar, Heba Elshahawi, Dina Naoum, Tarek Elsahrawy, Yomna Elhawary

**Affiliations:** 1 Department of Neurology and Psychiatry, Faculty of Medicine, Ain Shams University, Cairo, Egypt; 2 Al Mashfa Hospital, Cairo, Egypt

**Keywords:** suicidality, borderline personality disorder, Egyptian, impulsivity, psychiatric comorbidity, risk factor, suicide attempts

## Abstract

**Background:**

Addressing the risk of suicidality in borderline personality disorder (BPD) is a crucial issue. The notion that impulsive individuals are more likely to plan for suicide attempts is important for many reasons in both theoretical and clinical decision-making.

**Aim:**

The aim of this study was to investigate potential risks of suicidality in BPD and to correlate it to impulsivity.

**Setting:**

The study was conducted at the Institute of Psychiatry, Ain Shams University and Al Mashfa Private Hospital.

**Methods:**

Ninety-one participants were included in the study: 30 patients were diagnosed as BPD without axis I comorbidities, 31 BPD patients had psychiatric comorbidities and 30 healthy subjects were assessed by using Structured Clinical Interview for DSM-IV Axis I Disorders (SCID-I), Structured Clinical Interview for DSM-IV Axis II Disorders (SCID-II), Suicide Behavior Questionnaire-Revised (SBQ-R), the Arabic version of Barratt’s Impulsiveness Scale-11 (BIS-11) and Global Assessment of Functioning scale.

**Results:**

There was a significant difference in suicidality using the SBQ-R between the healthy controls and BPDs without and with comorbidities. Healthy controls showed low suicidality in only 3.3%, while it was higher in both groups of BPD. The total score of BIS was as follows: 62.5 (±10.1 SD) in group A, 79.4 (±12 SD) in group B and 80.3 (±12.5 SD) in group C, which denote mild, moderate and moderate-to-severe degree of impulsivity in group A, B and C, respectively. Suicidality was positively correlated with (AI item: lack of span Attention in Attentional Facet) (*r* = 0.489, *p* = 0.006), (PI item: lack of self-control in planning facet) (*r* = 0.401, *p* = 0.028), as well as (MII item: lack perseverance in holding off impulsive acts in motor facet) (*r* = 0.471, *p* = 0.009).

**Conclusion:**

Proper assessment of associated psychiatric comorbidities and impulsivity among BPD patients will help preventing of future suicidal attempts.

## Introduction

Borderline personality disorder (BPD) is the most frequent personality disorder, reaching approximately 15% – 50% in psychiatric inpatients and 11% in psychiatric outpatients.^1^ The limited epidemiological data available on BPD suggest that the prevalence of the disorder is between 0.2% and 1.8% in the general community. No data on the incidence rate of new cases of the disorder have been reported, and inferences about incidence based on prevalence rates are complicated by differences in the formal designation of personality disorders before and after DSM-III was issued.^2^ It is a serious mental health problem that is associated with a considerable degree of psychosocial impairment and high rate of suicide reaching (10%).^1^

The rate of suicide in patients with BPD is estimated to be between 8% and 10%, much greater than that in the general population.^3^ However, 60% – 70% of patients with BPD attempt suicide; those unsuccessful suicide attempts are far more frequent than completed suicides.^4,5^

Moreover, BPD coexists with depression, anxiety and substance abuse. Symptoms of these complicated conditions may mislead the clinician to the right diagnosis of personality disorder entirely. Patients with BPD (including those with comorbid depression) have reported greater lethality for suicide attempts than those with depression alone.^5^

The history of prior attempts is among the most powerful predictor of completed suicide while only 10% – 15% of attempters become completers; 30% – 40% of cases with completed suicide have a history of prior attempts. Most repetitive attempts occur in young women and decrease with time.^6^

The research literature on the management of suicidality in BPD does not provide evidence-based guidelines for the prevention of death by suicide.^7^ Thus, the need to implement effective prevention and treatment interventions in the population of the BPD patient against suicide is essential, in order to decrease their morbidity and mortality rates.^8^

Impulsivity, which is a frequently misunderstood aspect of suicide risk, has long been considered important to the aetiology and prediction of suicide. Some research suggests that impulsivity is maladaptive: has been linked to personality disorders, substance abuse and criminality.^9^

In Egypt few studies about impulsivity among patients with BPD were conducted^10^; furthermore, no previous studies addressed its relation to suicidality.

Therefore, the aim of this study is to identify suicidality and impulsivity among patients with BPD associated with or without axis I comorbidity and to further find its correlation to impulsivity and suicidality.

## Methods

### Study design

The current study is a comparative cross-sectional study, whereas a convenient sample was taken.

### Setting

The study was conducted in two psychiatric hospitals located in Cairo, Egypt, which are the Okasha Institute of Psychiatry (Ain Shams University) and Al Mashfa Private Hospital. They represent both the low-middle and high socio-economic class, respectively.

### Study population and sampling

Patients were eligible for the study if they fulfilled DSMIV diagnostic criteria for BPD, and their age was between 18 and 60 years. Patients were ineligible if they had any SCID I or SCID II diagnosis disorder, received recent electroconvulsive therapy, or have associated chronic medical or neurological disorder.

Controls were chosen from healthy employees and their relatives in both hospitals, and they did not have any axis I or II psychiatric comorbidities.

Sample size calculation: Assuming a high proportion of BPD patients with history of suicidal attempts (50%) and only a small proportion of healthy people having history of suicidal attempts (5% or less), it is estimated that 60 patients and 30 controls will achieve more than 90% power to detect difference between two groups setting alpha error at 0.05.

The programme used for sample size calculation is the Epi Info 7 programme.

### Operational definition

*Rigid family*: Authoritarian leadership, which controls behaviour with strict rules and roles.

*Structured family:* Rules and roles are where everyone is aware and on board. This empowers children to help create rules and roles when appropriate. This creates individual autonomy.

*Flexible family*: Rules, roles and decision-making are susceptible to change when situations warrant. The power dynamics are shared when appropriate.

*Diffused family*: Chaotic, and erratic rules and roles, ineffective and confusing leadership are dominant.

### Tools and procedures

All subjects (cases and controls) who accepted to participate in the study were interviewed using the Arabic Version of SCID II (Structured Clinical Interview for DSM-IV Axis II Disorders)^11,12^ and the Arabic version of SCID I (Structured Clinical Interview for DSM-IV Axis I Disorders).^13,14^

Sixty-seven subjects diagnosed as BPD by SCID II were selected. Out of these 67 patients of borderline, six of them were not included in the study, as they withdrew their consent. The remaining 60 patients were further subdivided into: 30 patients without Axis I comorbidities (group B) with a mean age of 28.9 years (SD ± 5.5) and 31 patients with Axis I comorbidities (group C) with a mean age of 26 years (SD ± 6.8).

Thirty healthy subjects matched for age and sex were included as a control group (group A); they were chosen from the employees and their relatives of both hospitals.

All participants (cases and controls) were asked to complete the following tools:

1. **SBQR: Suicide Behavior Questionnaire-Revised (SBQ-R)^15^:** It is a self-administered questionnaire assessing suicide behaviours. It has four items, each tapping a different dimension of suicidality. Sum of the scores should range from 3 to 18.

Item 1 taps into lifetime suicide ideation and/or suicide attempt; item 2 assesses the frequency of suicidal ideation over the past 12 months; item 3 assesses the threat of suicide attempt; item 4 evaluates self-reported likelihood of suicidal behaviour in the future.

The test has a sensitivity of 93% and specificity of 95%, while cutoff scores in adult patients are equal or more than 8. Reliability values of the Arabic version were found to be high as well (Cronbach’s alpha = 0.88).

2. **Barratt Impulsiveness Scale, version 11 (BIS-11): Arabic version^16,17^**: It is a self-administered questionnaire designed to assess the personality/behavioural construct of impulsiveness. It includes 30 items that yield six first-order factors (attention, motor, self-control, cognitive complexity, perseverance and cognitive instability impulsiveness) and three second-order factors (attentional, motor and non-planning impulsiveness).

Its scoring is as follows: 60–70 is mild, 70–80 is moderate and if more than or equal to 80 then impulsivity is severe. Total scoring of Barratt’s scoring: 30–120, (AI): 5–20, (AII): 3–12, (MI): 7–28, (MII): 4–16, (PI): 6–24, (PII): 5–20.

Low scores on both (AI) and (AII) show good attention span, cognitive stability and the qualities of non-impulsivity. Low scores on both (MI) and (MII) show good control of motor actions and persevere in holding off on impulsive actions. Low scores on both (PI) and (PII) show good self-control in planning for future and possessing good cognitive ability for complexity, which is the reverse of an impulsive attitude.

3. **Global Assessment of Functioning^18^:** It is a numeric scale (0 through 100) used by mental health clinicians and physicians to rate subjectively the social, occupational and psychological functioning of adults.

### Statistical analysis

All analyses were carried out using SPSS, version 20 (SPSS Inc., Chicago, Illinois, USA). The results were tabulated and statistically analysed using the following tests: Student’s *t* test and the *c*^2^ test. ANOVA test and post hoc analysis were used to compare values between three groups. Furthermore, Spearman correlation was used to test the association between suicidality, impulsivity, Global Assessment of Functioning (GAF) and socio-economic state of patients. Significance level was set at *p* < 0.05.

### Ethical considerations

The FMASU REC is organised and operated according to guidelines of International Council on Harmonization (ICH) and the Islamic Organization for Medical Services (IOMS), the United States Office for Human Research Protections and the United States Code of Federal Regulations and operates under Federal Wide Assurance number: FWA 000017585. Patients were informed about the nature of the research and the confidentiality of the obtained information.

## Results

### Socio-demographic data and family circumstances

There was significant difference between the three groups with regard to age (*p* = 0.002) and the occupation (*p* < 0.001).

Family role differed significantly between the three groups; structured family was observed more in group A (43.3%), while diffused family role was more evident in group B (40%) and group C (45.2%), respectively, with *p*-value 0.007.

Furthermore, the present history of abuse differed significantly between the three groups (*p* = 0.015), which nearly quadrupled in group B 53.3% (*n* = 16) and group C 51.6% (*n* = 16) in comparison to group A 13.3% (*n* = 4). Emotional abuse was the most evident type of abuse in the three groups.

Table 1 clarifies that borderline personality subjects were significantly exposed to perpetration from their first degree family member (*p* = 0.005).

**TABLE 1:** Socio-demographic and family circumstances.

Variable	Controls 30		BPD 30		BPD with axis I 31		*p*
	*n*	%	*n*	%	*n*	%	
**Gender**							0.053
Male	11	36.7	3	10.0	8	25.8	
Female	19	63.3	27	90.0	23	74.2	
**Occupation**							< 0.001*
Professional	25	83.3	16	53.3	8	25.8	
Skilled	1	3.3	3	10.0	3	9.7	
Unemployed	4	13.3	11	36.7	20	64.5	
**Marital status**							0.089
Single	12	40.0	18	60.0	22	71.0	
Married	15	50.0	11	36.7	6	19.4	
Separated	2	6.7	0	0.0	1	3.2	
Divorced	1	3.3	1	3.3	2	6.5	
**Social class**							0.132
Low	15	50.0	19	63.3	14	45.2	
Middle	13	43.3	5	16.7	12	38.7	
High	2	6.7	6	20.0	5	16.1	
**Parental separation**							0.078
Yes	4	13.3	11	36.7	11	35.5	
No	26	86.7	19	63.3	20	64.5	
**Family role**							0.007
Rigid	4	13.3	4	13.3	9	29	
Structured	13	43.3	5	16.7	3	9.7	
Flexible	9	30	9	30	5	16.1	
**Present history of abuse**							0.002
Yes	4	13.3	16	53.3	16	51.6	
No	26	86.7	14	46.7	15	48.4	
**Past history abuse**							0.003
Yes	8	26.7	18	60.0	14	45.2	
No	22	73.3	12	40.0	17	54.8	
**Perpetrator**							0.005
No	21	70	10	33.3	10	32.3	
Partner	2	6.7	3	10	1	3.2	
Fir**st degree** Family member	7	23.3	12	40	18	58.1	
Stranger	0	0	5	16.7	2	6.5	

BPD, borderline personality disorder.

### Suicidality among the studied groups

There was a statistically significant difference between the two groups of borderline personality in total mean score, item 2 which denotes the frequency of suicidal ideation over the past 12 months and item 4 showing the likelihood of suicidal behaviour in the future, which means that suicidality was found to be significantly more prevalent in borderline personality with comorbidity (group C) than borderline personality without comorbidity (group B) (54.8% in group C vs 36.7% in group B).

There was significant difference between the three groups regarding total score and sub-items of Baratt’s impulsiveness scale. There was high significant difference between the three groups regarding the total score of Barratt’s impulsiveness scale of Barratt (AII), Barratt (MI) and Barratt (PI) with *p* < 0.001. The difference between the three groups regarding Barratt (MII) and Barratt (PII) was significant with *p* = 0.049 and *p* = 0.046, respectively.

On post hoc test, there was significant difference between group A and B regarding Baratt’s (AII), Baratt’s (MI) and Baratt’s (PI). All items of Barrat’s impulsiveness scale increased in group C versus group A. On the other hand, when comparing the two groups of bipolar with each other (group B and C), Baratt’s (PII) was increased in group C.

Significant difference was found in GAF mean score when comparing the three groups. Highest scores of GAF were in controls (group A), then in BPD group (Group B), while the lowest scores were in BPD cases with comorbidities (group C).

Moreover, we compared, in our study, the GAF scores in association with suicidality. It showed highly significant results where the mean score of GAF in BPD cases with low suicidality was higher than those with high suicidality (Table 2).

**TABLE 2:** Suicidality and impulsivity among the three studied groups.

Variable	Group A (healthy control)1		Group B (BLP)2 without comorbidity		Group C (BLP with comorbidity)3		ANOVA test	Group A vs. Group B	Group A vs. Group C	Group B vs. Group C
	Mean	±SD	Mean	±SD	Mean	±SD	*p*	Post hoc analysis		
**Suicidality: Suicide Behavior Questionnaire-Revised (SBQ-R)**										
SBQ-R (T): Total score	4.2	±1.7	7.1	±4.3	9.2	±4.9	< 0.001	Sig.	Sig.	Sig.
SBQ-R (1) Item 1 taps into lifetime suicide ideation and/or suicide attempt	1.5	± 0.8	2.1	±1.1	2.6	±1.2	0.001	Sig.	Sig.	NS
SBQ-R (2) Item 2 assesses the frequency of suicidal ideation over the past 12 months	1.3	±0.7	2.0	±1.4	2.7	±1.5	< 0.001	Sig.	Sig.	Sig.
SBQ-R (3) Item 3 assesses the threat of suicide attempt	1.1	±0.4	1.6	±0.9	2.0	±1.1	< 0.001	Sig.	Sig.	NS
SBQ-R (4) Item 4 self-reported likelihood of suicidal behaviour in the future	0.3	±0.4	1.3	±1.5	2.0	±1.5	< 0.001	Sig.	Sig.	Sig.
**Impulsivity: Barratt Impulsiveness Scale, version II (BIS-II)**										
Barratt total score	62.5	±10.1	79.4	±12	80.3	±12.5	< 0.001	Sig.	Sig.	NS
Barratt (AII)	6.4	±2.6	8.7	±2.4	8.3	±2.5	< 0.001	Sig.	Sig.	NS
Barratt (MI)	15.2	±4.1	20.7	±5	18.5	±6.2	< 0.001	Sig.	Sig.	NS
Barratt (MII)	7.6	±2.9	8.9	±2.1	9.3	±2.9	0.049	NS	Sig.	NS
Barratt (PI)	11.5	±3	16.3	±4.7	16.7	±4	< 0.001	Sig.	Sig.	NS
Barratt (PII)	11.5	±3.2	11.5	±3.3	13.4	±3.4	0.046	NS	Sig.	Sig.
**Global Assessment of Functioning**										
GAF	94.7	±1.9	79.7	±13.3	66.0	±19	< 0.001	Sig.	Sig.	Sig.

SBQ-R, Suicide Behavior Questionnaire-Revised; Sig., significant; NS, non significant; BLP, Borderline personality; ANOVA, Analysis of variance.

Barratt: Low scores on both (AI) and (AII) show good attention span, cognitive stability and the qualities of non-impulsivity. Low scores on both (MI) and (MII) show good control of motor actions and persevere in holding off on impulsive actions. Low scores on both (PI) and (PII) show good self-control in planning for future and possessing good cognitive ability for complexity.

### Correlation between suicidality and impulsivity

Among the BPD without axis I comorbidities (group B), suicidality (as measured by SBQ-R) was significantly correlated to most of the impulsivity scores that was measured by BIS-R (Table 2). Suicidality was positively correlated with AI item: lack of span Attention in Attentional Facet (*r* = 0.489, *p* = 0.006), PI item: lack of self-control in planning facet (*r* = 0.401, *p* = 0.028), as well as MII item: lack perseverance in holding off impulsive acts in motor facet (*r* = 0.471, *p* = 0.009) of Barratt’s impulsiveness score (BIS-R), while a strong negative correlation was found between SBQ-R items and GAF (*r* = -0.671, *p* < 0.001). When we studied the association between suicidality measured by SBQ-R scale and level of impulsivity shown by BIS-11 scale, all the results related to the group of BPD without Axis I comorbidities showed no statistical significance using the *t* test. Total score of Barratt’s impulsiveness score was mildly lower (mean = 77.58) with (11.07 ± SD) in those who had low suicidality by SBQ than those who were high in suicidality (mean = 82.64) with (13.32 ± SD).

Among the BPD with axis I comorbidities (group C), results show total score of Barratt’s impulsiveness score was mildly higher (mean = 80.86) with (10.86 ± SD) in those who had low suicidality by SBQ than those who were high in suicidality (mean = 79.76) with (14.03 ± SD) which is the opposite of what was found in the group of BPDs without Axis I comorbidities.

Suicidality showed negative correlation between total score of SBQ-R with (PII item: lack of cognitive complexity in nonplanning facet) of BIS-R (*r* = –0.394, *p* = 0.028), and negative correlation between item 2 in SBQ-R and (PII item) in BIS-R (*r* = –0.483, *p* = 0.006), while it showed no correlation with GAF (Table 3).

**TABLE 3:** Spearman correlation between Suicide Behavior Questionnaire-Revised and Barratt’s Impulsiveness Scale-11 in borderline personality disorder with axis I comorbidities.

BLP comorbidities	Barratt (AI)	Barratt (AII)	Barratt (MI)	Barratt (MII)	Barratt (PI)	Barratt (PII)
**SBQ-R(T)**						
*rs*	0.010	0.116	0.172	0.104	−0.098	−0.394
*p*	0.958	0.534	0.356	0.577	0.601	0.028
**SBQ-R(1)**						
*rs*	0.044	0.177	0.182	0.100	0.003	−0.289
*p*	0.813	0.342	0.327	0.594	0.989	0.114
**SBQ-R(2)**						
*rs*	0.018	0.020	0.154	0.019	−0.222	−0.483
*p*	0.923	0.915	0.407	0.919	0.229	0.006
**SBQ-R(3)**						
*rs*	0.217	0.200	0.307	0.156	0.093	−0.209
*p*	0.240	0.281	0.093	0.402	0.617	0.260
**SBQ-R(4)**						
*rs*	−0.136	−0.006	0.083	0.096	−0.157	−0.382*
*p*	0.467	0.976	0.658	0.609	0.399	0.034

SBQ-R, Suicide Behavior Questionnaire-Revised; BLP, Borderline personality.

* , statistical significance.

## Discussion

The present study is one of the preliminary studies in Egypt which was interested in exploring the prevalence of suicide among an Egyptian sample of BPD and determines its socio-demographic and clinical correlates.

The rate of completed suicide in patients with BPD in clinical populations is estimated to be between 8% and 10%. However, about 60% – 70% of patients with BPD attempt suicide^3^ that is similar to our results by using the SBQ-R; suicidality among the BPD is estimated to be 36.7% in BLP and 54.8% in BPD with psychiatric comorbidity. The obtained result is slightly lower than what Oldham^20^ stated in his results that 60% – 70% of patients with BPD attempt suicide and this might be explained by the difference in sample size and assessment methods.^19^

Several studies suggest that comorbidity is associated with worse clinical status in BPD than for the single diagnosis, as comorbidity plays a major role in suicide progression and indicates a higher suicidal risk.^20^ The same concept is proved in our results that suicidality was found to be significantly more prevalent in borderline personality with comorbidity (group C) than borderline personality without (group B), with significant difference regarding the total score of SBQ-R, the frequency of suicidal ideation over the past 12 months and the likelihood of suicidal behaviour in the future.

The claim that impulsivity has its role in facilitating suicidal actions among those with suicidal ideation is supported by our results among the BPD without comorbidity as mentioned in Table 2.^2^ Suicidality was positively correlated with different items of Barratt’s impulsiveness scale.

Bryan and Rudd stated that impulsivity ‘may actually be a more significant indicator of suicide attempt than the presence of a specific suicide plan’.^21^ Furthermore, Barker et al.^10^ showed that there is a clear support for some role of impulsivity in suicidal behaviour. These findings could be consistent with our findings, if we compare BPD cases with and without comorbidities with healthy controls scores as shown earlier.^22^

However, it was surprising that the role of impulsivity has failed to support the claim of increased suicidality among BPD with comorbidities, as suicidality showed negative correlation between total scores of SBQ-R. The presence of BPD and other axis I disorder may suggest that these pathologies contribute more to suicidality than impulsivity alone.^23^

Meanwhile, a relation has been found between increased suicidal behaviour and comorbidity of substance abuse disorder with BPD as Oldham^20^ examined the prognostic significance of comorbid substance abuse in patients with BPD. The patients were followed prospectively over a 7-year period. These researchers found that patients with comorbid substance abuse and BPD perceived themselves to be at significantly more risk for suicide than did the comparison groups of patients having BPD without comorbidity.^20^

These findings come in agreement with other findings; Baca Garcia et al. (2001)^23^ found that impulsivity of the suicide attempt was negatively related to the lethality of the attempt. Furthermore, anecdotal accounts provide evidence that even impulsive individuals frequently spend a great deal of time carefully planning their suicide attempts. These findings converge with the predictions of Joiner’s theory which proposes that impulsivity is a distal risk factor for suicide, which operates via the exposure of impulsive people to painful and provocative experiences. They do not necessarily attempt suicide without prior planning and that is why their attempts tend to be less medically serious.^24^

In our study, significant differences were found in GAF mean score on comparing three groups with the lowest scores were in BPD cases with comorbidity. The wide difference shows how this group is heterogeneous.

## Clinical implications

Although our study is one of the preliminary studies in Egypt that is interested in exploring the effect of Axis I comorbidities upon the risk assessment (Impulsivity and suicidality) of BPD Egyptian patients in comparison to those patients without comorbidities, an important limitation of this study lies in the difficulty of generalising the data because of the small sample size. Therefore, more research should be performed on larger samples. Another limitation is the need to evaluate of these subjects over a longitudinal period of time to determine the effect of these comorbidities upon their prognosis. In addition, the control group was 30 and was recruited from healthy hospital employees.

## Conclusion

Borderline personality disorder is a rising problem in our country with high suicidality. Comorbid conditions and impulsivity were considered factors that significantly correlate with suicidality, which could be prevented early, diagnosed and properly treated to decrease the burden of the emotional pain of these patients.
